# Geranylgeranoic acid and the MAOB–CYP3A4 axis: a metabolic shift underlying age-related liver cancer risk

**DOI:** 10.3389/fragi.2025.1680031

**Published:** 2025-10-09

**Authors:** Yuki Tabata

**Affiliations:** Nutritional Biochemistry, Faculty of Wellness Studies, Department of Nutritional Health, Kwassui Women’s University, Nagasaki, Japan

**Keywords:** geranylgeranoic acid, monoamine oxidase B, cytochrome P450 3A4, hepatocellular carcinoma, aging, metabolic turning point, lipid biomarker, oxidative stress

## Abstract

Geranylgeranoic acid (GGA) is a naturally occurring acyclic isoprenoid with chemopreventive effects against hepatocellular carcinoma. In mammals, GGA is endogenously synthesized via the oxidative metabolism of geranylgeraniol by monoamine oxidase B (MAOB). However, MAOB activity decreases with age, leading to reduced hepatic GGA levels. Emerging evidence suggests that cytochrome P450 3A4 (CYP3A4) may compensate for this decline, providing an alternative oxidative pathway in MAOB–deficient conditions. This mini-review summarizes the current findings on GGA biosynthesis and metabolism in the aging liver, focusing on the MAOB–CYP3A4 axis, in which MAOB serves as the primary enzyme for endogenous GGA synthesis and CYP3A4 provides a compensatory pathway under MAOB–deficient conditions, and its relevance to age-related hepatic dysfunction. By discussing recent evidence on enzymatic compensation and age-dependent metabolic changes, this review highlights how the CYP3A4–GGA pathway may help unravel the complexity of hepatic aging. These findings may provide a mechanistic basis for developing preventive strategies targeting age-related hepatocarcinogenesis, particularly in older individuals with reduced MAOB–GGA activity.

## 1 Introduction

Hepatocellular carcinoma (HCC) remains a global health burden, ranking as the third leading cause of cancer-related deaths worldwide (Sung et al., 2021). Its development involves a complex interplay between genetic, metabolic, and environmental factors. In recent years, increasing attention has been directed toward metabolic regulators in the liver, particularly those involved in lipid metabolism and oxidative stress responses (Elhinnawi et al., 2025; Kodama and Takehara, 2024). Among these, monoamine oxidase B (MAOB) has emerged as a noteworthy player due to its involvement in the endogenous synthesis of geranylgeranoic acid (GGA), a lipid mediator with potential tumor-suppressive properties (Tabata et al., 2021; Tabata and Shidoji, 2020). GGA, an acyclic isoprenoid, has demonstrated antitumor effects, particularly in the liver (Nakamura et al., 1995; Muto et al., 1996; Muto et al., 1999).

It is synthesized via the oxidation of geranylgeraniol by MAOB, underscoring the enzyme’s potential protective role. GGA has been reported to induce pyroptotic cell death in precancerous hepatic cells via Toll-like receptor 4 (TLR4) activation (Shidoji, 2023; Shidoji, 2024; Yabuta and Shidoji, 2020). This process involves ER stress responses and activation of caspase-4 and caspase-1, leading to gasdermin D–mediated pyroptosis. Importantly, pharmacological inhibition or siRNA knockdown of TLR4 completely suppresses these effects, indicating that TLR4 plays an essential upstream role in GGA-induced cell death (Shidoji, 2023). Collectively, these findings suggest that the GGA–TLR4 axis, together with MAOB-derived GGA, contributes to hepatic immune surveillance by eliminating premalignant cells through pyroptosis.

However, the role of MAOB in hepatic physiology appears temporally regulated. With aging, the expression and activity of MAOB decline, resulting in reduced hepatic GGA levels (Tabata et al., 2021). This reduction coincides with an increased risk of spontaneous hepatocarcinogenesis in certain mouse models, such as C3H/HeN mice (Tabata et al., 2021). The idea that age-dependent changes in MAOB and GGA may underlie the shift from a tumor-suppressive to a tumor-permissive hepatic microenvironment introduces a compelling paradigm for metabolic carcinogenesis. This mini-review addresses the concept that MAOB serves as a gatekeeper enzyme for hepatic tumor suppression by facilitating GGA production. The discussion focuses on the metabolic changes that occur with aging, its consequences for GGA availability, and the compensatory role of cytochrome P450 3A4 (CYP3A4), a hepatic enzyme that may partially substitute for MAOB in GGA synthesis (Tabata and Shidoji, 2022) but also potentially promotes tumor progression (Mohamed et al., 2024). By analyzing the MAOB–GGA–CYP3A4 axis, this review seeks to underscore a critical yet underexplored dimension of liver tumor biology and propose experimental directions for future investigations.

Beyond their potential roles in liver tumor biology, both MAOB and CYP3A4 are well-characterized enzymes with established physiological functions in non-cancer contexts. MAOB is widely recognized for its role in the oxidative deamination of neurotransmitters and trace amines, contributing to neuromodulation and cellular redox balance, and it has also been extensively studied as a therapeutic target in neurodegenerative diseases such as Alzheimer’s disease and Parkinson’s disease (Asim et al., 2025; Junkes et al., 2025; Mohan et al., 2025). Similarly, CYP3A4 is one of the most abundant hepatic cytochrome P450 enzymes, responsible for the metabolism of approximately half of all clinically used drugs and several endogenous substrates including steroids and bile acids (Zhang et al., 2024; Zanger and Schwab, 2013).

GGA, an endogenous isoprenoid metabolite with known tumor-suppressive effects in the liver, has been proposed as a potential lipid-based biomarker of tumor susceptibility. This review examines the age-dependent regulation of hepatic GGA levels via MAOB and CYP3A4 pathways and assesses its relevance to age-related cancer biology.

## 2 MAOB and GGA: a hepatic tumor suppressor pathway

GGA was initially synthesized as a potential pharmaceutical agent, and its antitumor properties, particularly its ability to induce cell death in HCC cells, were recognized in the early stages of its development (Muto et al., 1996; Muto et al., 1999; Araki et al., 1995). While it was initially approached as a synthetic compound, its biological significance broadened dramatically when Shidoji and Ogawa later discovered that GGA occurs naturally in certain medicinal plants, including turmeric (Tabata, 2025; Shidoji and Ogawa, 2004). This pivotal finding reframed GGA as not only a drug candidate but also a bioactive lipid with potential physiological relevance. Building upon this discovery, Shidoji et al. extended this investigation to mammals. Shidoji and colleagues further demonstrated that GGA is not only present in plant-derived foods but is also endogenously synthesized in mammals, including humans (Mitake and Shidoji, 2012; Endo et al., 2011; Shidoji and Tabata, 2019). This shift in understanding established GGA as a physiologically relevant metabolite, rather than merely an exogenous phytochemical.

**FIGURE 1:**
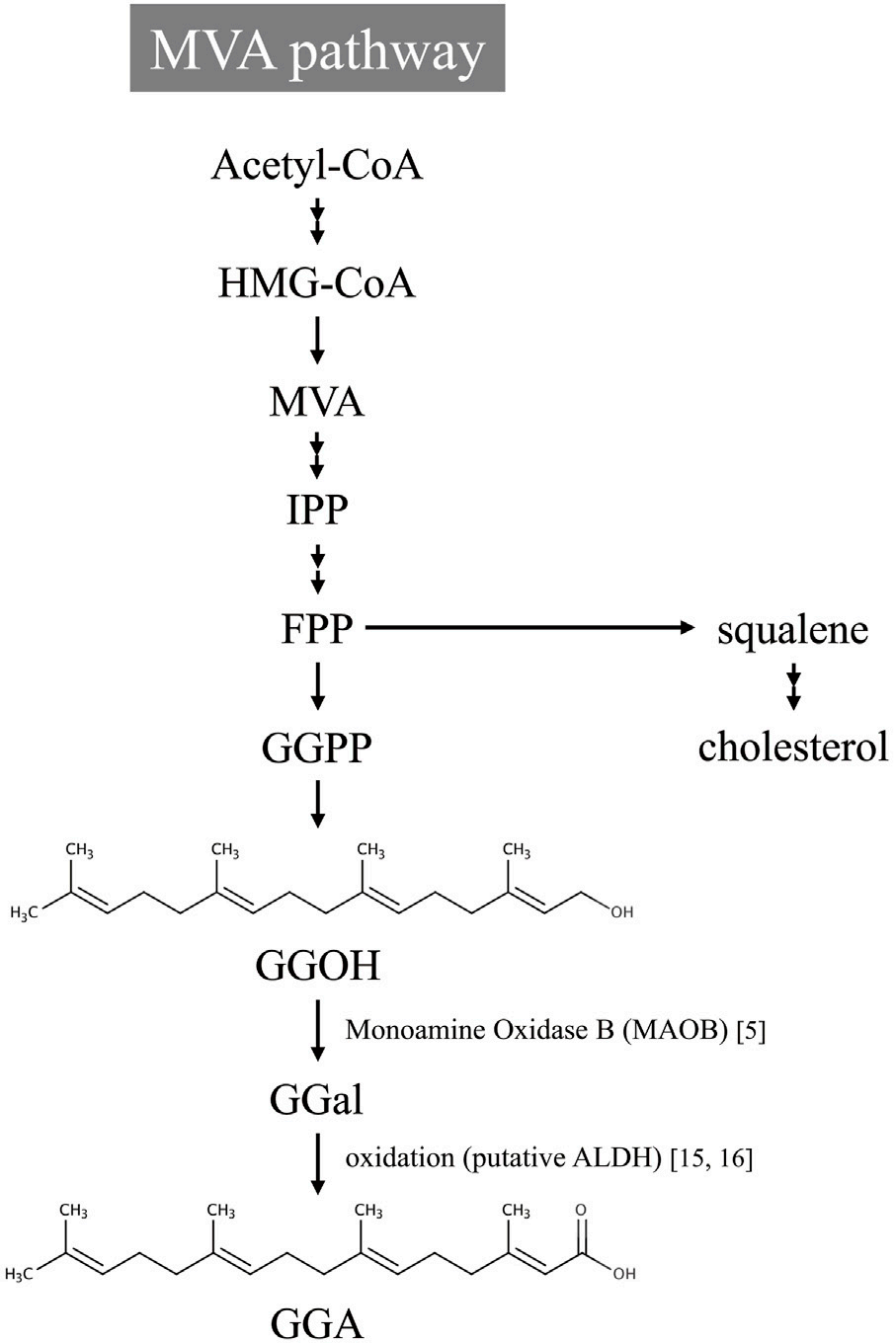
Proposed biosynthetic pathway of geranylgeranoic acid (GGA) from the mevalonate (MVA) pathway. Acetyl-CoA is metabolized through the MVA pathway to produce isopentenyl pyrophosphate (IPP), which is subsequently converted into farnesyl pyrophosphate (FPP). FPP serves as a branch point leading to both cholesterol biosynthesis and the formation of geranylgeranyl pyrophosphate (GGPP). GGPP is then dephosphorylated to geranylgeraniol (GGOH), which is oxidized to geranylgeranial (GGal) by monoamine oxidase B (MAOB). GGal is further oxidized, possibly via aldehyde dehydrogenase (ALDH) activity, and finally metabolized into geranylgeranoic acid (GGA).

Subsequent investigations have revealed that the biosynthesis of GGA in mammals involves MAOB, a flavin-containing enzyme traditionally recognized for its role in neurotransmitter metabolism (Müller and Möhr, 2019). MAOB catalyzes the oxidative deamination of geranylgeraniol, an intermediate of the mevalonate (MVA) pathway, to produce GGA (Tabata and Shidoji, 2020). This biosynthetic route is illustrated in Figure 1, which outlines the MVA pathway leading to GGA formation via MAOB-mediated oxidation of geranylgeraniol. The involvement of MAOB in this pathway positions the enzyme as a critical metabolic node linking lipid metabolism to cancer prevention. Recent studies have further expanded this concept by incorporating evidence from both endogenous and dietary sources of GGA, its metabolic regulation, and its tumor-suppressive mechanisms in hepatic tissues.

The relationship between age-related changes in GGA metabolism and hepatocarcinogenesis has been explored using C3H/HeN mice, a well-established model of spontaneous HCC (Tabata et al., 2021). In this model, hepatic GGA levels were found to decline markedly with age. This reduction was accompanied by a parallel, age-dependent decrease in the expression of MAOB, the key enzyme responsible for GGA biosynthesis (Tabata et al., 2021). Notably, this metabolic decline coincided with a period of increased susceptibility to hepatocarcinogenesis, suggesting that diminished GGA levels may represent a “metabolic turning point” that facilitates the initiation of hepatic tumor development.

Interestingly, a single oral administration of GGA or its 4,5-didehydro derivative during this metabolic turning point has been shown to suppress the progression of pre-neoplastic hepatocytes and significantly reduce the subsequent development of liver tumors (Tabata et al., 2021; Omori et al., 2023). The antitumor effect was most pronounced when GGA was administered at 11 months of age, while administration at 8 or 13 months yielded minimal or no effect (Tabata et al., 2021; Omori et al., 2023). These findings indicate that the tumor-suppressive function of GGA is temporally constrained, exerting its effects most effectively during a critical early phase of hepatocarcinogenesis.

Furthermore, several studies have reported that GGA induces HCC cell death via TLR4-mediated pyroptosis (Yabuta and Shidoji, 2020), suggesting an immune-mediated mechanism for the elimination of aberrant hepatocytes. Accordingly, the age-associated downregulation of MAOB, and the resultant loss of hepatic GGA, may represent not only a metabolic alteration but also a disruption of the liver’s intrinsic tumor surveillance system.

While these findings underscore the tumor-suppressive role of GGA, they also raise an important question: how does the age-associated decline in MAOB expression establish a window of vulnerability that permits tumor initiation? The following section examines in greater detail how age-dependent reductions in MAOB activity may contribute to hepatic carcinogenesis.

## 3 Age-related decline in MAOB and its implications for hepatic carcinogenesis

The expression and enzymatic activity of MAOB in the liver are closely regulated by age, with significant implications for hepatic tumor biology. Previous studies, including our own, have demonstrated a marked decline in hepatic Maob mRNA levels during aging in mice (Tabata et al., 2021; Saura et al., 1994). This age-associated reduction in MAOB expression coincides with a decrease in hepatic levels of GGA, as observed in longitudinal analyses of C3H/HeN mice, a strain predisposed to spontaneous hepatocarcinogenesis. Given that MAOB is the principal enzyme responsible for endogenous GGA biosynthesis, its downregulation represents a pivotal event contributing to the loss of hepatic tumor-suppressive capacity. The onset of spontaneous HCC in aged C3H/HeN mice was temporally aligned with this decline in MAOB expression. Notably, the period between 9 and 13 months of age appears to constitute a vulnerable phase, during which GGA levels drop below the threshold required for effective tumor surveillance. This vulnerable phase, termed the “metabolic turning point” marks a shift in the hepatic microenvironment from a tumor-resistant to a tumor-permissive state. During this interval, hepatocytes harboring genetic or epigenetic alterations may escape immune-mediated clearance due to insufficient GGA production.

Our experiments demonstrated that a single oral administration of GGA at 11 months of age, precisely during the metabolic turning point, significantly suppressed the development of spontaneous liver tumors in C3H/HeN mice (Tabata et al., 2021). In contrast, GGA administration at 7 or 17 months of age failed to produce a comparable protective effect. These findings suggest that early neoplastic lesions may be silently established during this narrow window of MAOB insufficiency and that timely GGA supplementation can prevent their progression. Similar tumor-suppressive effects were observed with the 4,5-didehydro derivative of GGA in parallel experiments (Omori et al., 2023).

It is important to note that the decline in MAOB expression is gradual rather than abrupt (Tabata et al., 2021), which may account for the narrow temporal window during which GGA intervention is effective. This progressive reduction creates a transient phase of metabolic insufficiency, wherein GGA biosynthesis drops to sub-protective levels without being entirely abolished. The implications of this are profound: hepatocarcinogenesis may not result solely from external carcinogenic exposures or genetic mutations, but also from an intrinsic failure of the liver’s metabolic immune surveillance system.

**FIGURE 2:**
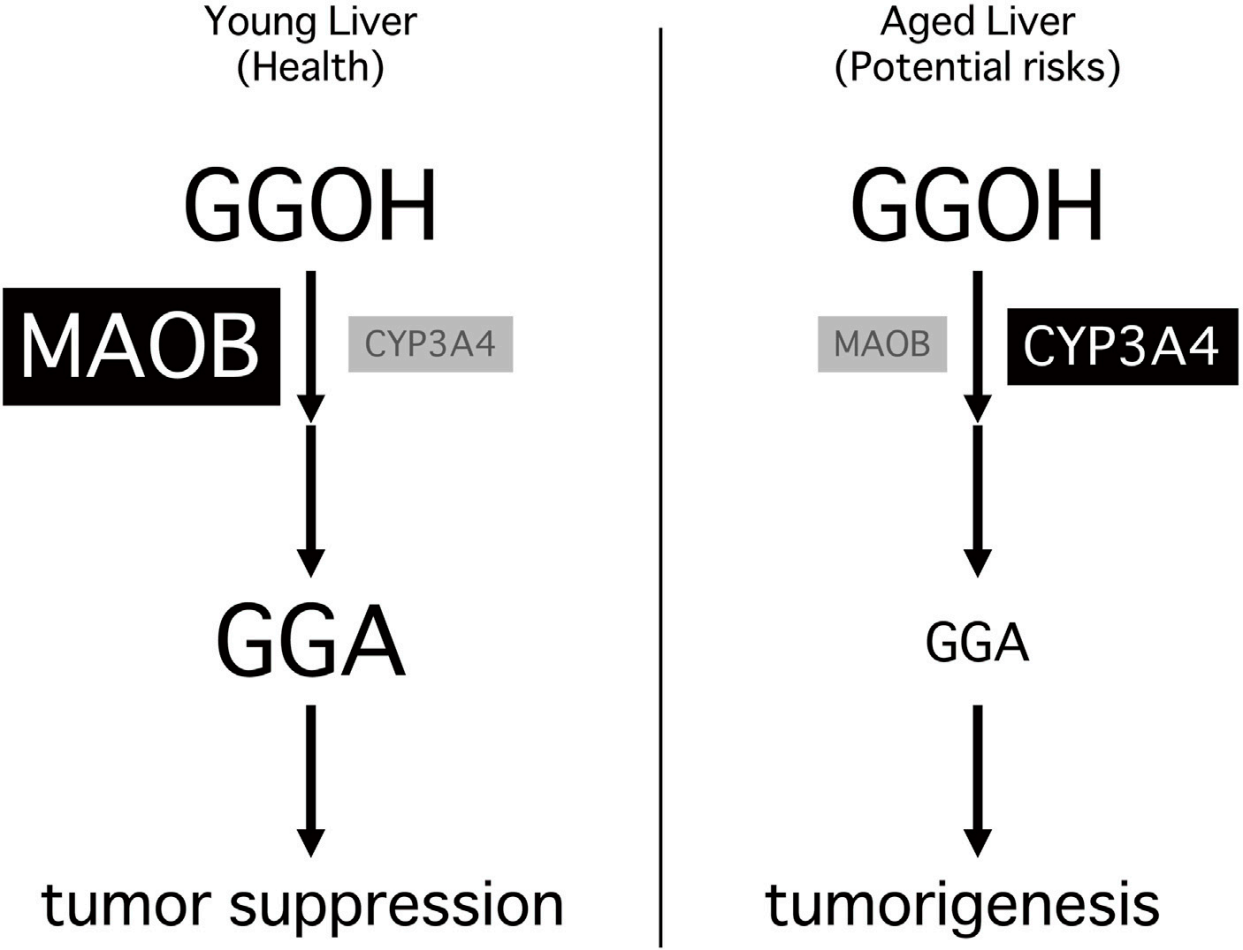
Age-dependent shift in hepatic GGA metabolism and its association with tumorigenesis. In young livers (left), monoamine oxidase B (MAOB) is highly expressed and efficiently converts geranylgeraniol (GGOH) to geranylgeranoic acid (GGA), which activates Toll-like receptor 4 signaling and contributes to tumor-suppressive homeostasis. In aged livers (right), the decline or loss of MAOB represents a metabolic turning point, leading to a marked reduction in hepatic GGA levels and the breakdown of tumor-suppressive homeostasis. Although cytochrome P450 3A4 (CYP3A4) partially compensates for this loss, the insufficient GGA levels fail to maintain adequate tumor suppression, resulting in a more tumor-permissive hepatic microenvironment and increased risk of hepatocellular carcinoma.

Taken together, the age-associated decline in MAOB expression and the consequent reduction in its downstream metabolite GGA establish a fundamental mechanistic link between aging and hepatocarcinogenesis. As illustrated in Figure 2, this age-dependent metabolic shift results in insufficient GGA biosynthesis, which is not fully compensated by CYP3A4 activity, ultimately impairing hepatic tumor surveillance. The “metabolic turning point” identified in this model represents a novel target for preventive intervention and may guide future strategies for early detection and chemoprevention in aging populations.

## 4 CYP3A4 and its dual role in the aging liver

As the age-related decline in MAOB compromises endogenous GGA production, the liver may activate compensatory oxidative pathways to maintain metabolic balance (Tabata and Shidoji, 2022). One such candidate is CYP3A4, a key member of the cytochrome P450 superfamily, which plays a pivotal role in xenobiotic metabolism, cholesterol regulation, all-trans-retinol oxidation, and oxidative biotransformation (Tian and Hu, 2014; Chen et al., 2000; Choi et al., 2021). Although CYP3A4 is predominantly recognized for its function in drug metabolism, emerging evidence suggests that it may also contribute to residual GGA biosynthesis from geranylgeraniol in MAOB-deficient conditions (Tabata and Shidoji, 2022).

Previous biochemical studies have demonstrated that CYP3A4 enzymes can catalyze the oxidation of isoprenoid alcohols such as farnesol and geranylgeraniol, resulting in the formation of their corresponding acids, including GGA. In our study, hepatic GGA synthesis was not completely abolished in MAOB-knockout cells; instead, the expression of CYP3A4 (or its murine orthologs) was upregulated, suggesting a compensatory role for this enzyme in GGA biosynthesis under MAOB-deficient conditions (Tabata and Shidoji, 2022). Therefore, in the context of age-associated MAOB depletion, CYP3A4 (or its murine orthologs such as Cyp3a11) may serve as a secondary pathway for GGA biosynthesis. In our previous study using C3H/HeN mice, hepatic GGA levels between 6 and 13 months of age were maintained at approximately 60% of the peak values observed in younger animals, suggesting that CYP3A4 may partially sustain GGA production during this period (Tabata et al., 2021).

However, this residual GGA production appears insufficient to exert robust tumor-suppressive effects, suggesting that CYP3A4-mediated compensation may operate below the threshold required for effective chemoprevention. Moreover, CYP3A4 activity is known to generate reactive oxygen species (ROS) as byproducts, particularly during the metabolism of xenobiotics (Özkan et al., 2021) and endogenous lipids (Tian and Hu, 2014). Chronic ROS accumulation can lead to DNA damage (Srinivas et al., 2019), lipid peroxidation (Wang et al., 2023), and cellular senescence (Gao et al., 2022), all of which are implicated in the promotion of hepatocarcinogenesis (Unsal and Belge-Kurutaş, 2017). Moreover, CYP3A4 plays a critical role in the metabolic inactivation of various chemotherapeutic agents, including sorafenib (Özkan et al., 2021) and other tyrosine kinase inhibitors widely used in the treatment of liver tumors (Teo et al., 2015). This enzymatic activity may reduce therapeutic efficacy and contribute to the development of drug resistance in HCC. In this context, elevated hepatic expression of CYP3A4 in aged or diseased individuals may inadvertently facilitate tumor progression rather than suppression. Thus, while CYP3A4 may partially compensate for GGA biosynthesis under MAOB-deficient conditions, this compensatory function comes at a potential cost, namely, increased oxidative stress and reduced responsiveness to chemotherapeutic agents. This dualistic nature, illustrated in Figure 2, raises critical questions about the enzyme’s overall role in age-related liver tumorigenesis. It is plausible that the timing and magnitude of CYP3A4 upregulation dictate whether its effects are beneficial or detrimental to the host. In the early stages of MAOB decline, modest CYP3A4 expression may sustain sufficient GGA levels to maintain immune surveillance. However, as aging progresses and CYP3A4 expression further increases, this balance may shift toward oxidative damage and tumor promotion. Thus, understanding the temporal dynamics of CYP3A4 expression and function in the aging liver is essential. Future studies should examine whether a “CYP3A4 threshold” exists, beyond which its tumor-promoting activities outweigh its compensatory benefits in GGA biosynthesis. Identifying such a tipping point could provide a valuable biomarker or therapeutic window for preventing age-related hepatocarcinogenesis.

## 5 Discussion: future perspectives and conclusion

The findings presented in this review offer new insights into the metabolic and molecular mechanisms underlying age-associated hepatocarcinogenesis. Central to our hypothesis is the functional axis comprising MAOB, GGA, and CYP3A4, which collectively define a hepatic metabolic landscape that shifts from a tumor-suppressive to a tumor-permissive state with advancing age. Understanding the dynamic interplay among these components is essential for identifying diagnostic biomarkers and developing targeted preventive strategies for HCC, particularly in aging populations.

One of the most pressing research priorities is to further elucidate the compensatory role of CYP3A4 and its murine orthologs (e.g., Cyp3a11) in GGA biosynthesis. Although existing evidence supports their involvement in maintaining residual GGA levels under MAOB-deficient conditions (Tabata and Shidoji, 2022), the enzymatic efficiency, substrate specificity, and regulatory mechanisms of this alternative pathway remain incompletely characterized. A quantitative assessment of Cyp3a11 expression in aging liver tissue, particularly across the critical period between 6 and 15 months of age in C3H/HeN mice, would be a valuable first step. Such data could clarify whether a “CYP3A4 threshold” exists, beyond which the enzyme’s pro-oxidative and potentially pro-oncogenic activities surpass its compensatory capacity for GGA synthesis.

Additionally, it is essential to investigate whether pharmacological or genetic modulation of CYP3A4 activity can influence the incidence or progression of spontaneous liver tumors in MAOB-deficient or aging animal models. For instance, selective inhibition of CYP3A4 in advanced age may attenuate oxidative stress and mitigate drug resistance, while early-phase activation could help sustain GGA biosynthesis during the initial decline of MAOB. These dual and context-dependent roles underscore the need for precisely timed and individualized therapeutic strategies targeting CYP3A4.

Another promising avenue of research lies in the validation of the MAOB–GGA–CYP3A4 axis in human liver tissue. Analyses of post-mortem liver samples or biopsy specimens from individuals across different age groups could elucidate age-related changes in MAOB and CYP3A4 expression, hepatic GGA concentrations, and markers of inflammation and fibrosis. Correlating these molecular findings with clinical parameters—such as tumor incidence, liver function, and therapeutic responsiveness—may provide a critical translational link between experimental models and patient outcomes. In addition, the application of high-throughput technologies, including single-cell RNA sequencing and spatial transcriptomics, could enable precise mapping of MAOB and CYP3A4 expression within distinct hepatic cell populations. These approaches may uncover cell-type-specific regulation of GGA metabolism and help identify the cellular compartments most crucial for tumor immune surveillance in the human liver.

Finally, the concept of a “metabolic turning point” proposed in this review may have broader implications beyond hepatocellular carcinoma. Age-related declines in lipid-derived mediators and their compensatory enzymatic pathways could represent a generalizable mechanism contributing to tumor susceptibility in other tissues. Investigating similar metabolic inflection points across diverse organ systems and cancer types may uncover shared age-associated vulnerabilities. Such insights could inform the development of systemic preventive strategies that target metabolic dysregulation as a common feature of aging-related oncogenesis.

Beyond these mechanistic insights, the MAOB–GGA–CYP3A4 axis may also offer practical implications for prevention and therapy. Dietary intake of GGA-rich foods or pharmacological analogs could be investigated as potential strategies to sustain hepatic GGA levels during aging. In addition, selective modulation of CYP3A4 activity may provide context-dependent benefits, with early activation helping to maintain GGA synthesis, whereas later inhibition could reduce oxidative stress and mitigate drug resistance. Moreover, hepatic or circulating GGA levels may serve as lipid-based biomarkers to guide individualized risk stratification and preventive interventions in geriatric oncology. Together, these translational perspectives highlight the potential of targeting GGA metabolism to support age-adapted cancer prevention and to bridge experimental insights with clinical applications.

Limitations. While this review highlights emerging insights into the MAOB–GGA–CYP3A4 axis and its role in age-related hepatocarcinogenesis, several limitations should be acknowledged. First, much of the current understanding relies on preclinical studies, and direct experimental validation in human liver tissue remains limited. Second, the mechanistic links between GGA, TLR4 activation, and hepatic tumor suppression, although supported by experimental evidence, require further confirmation across different models and contexts. Third, potential confounding factors such as dietary patterns, lifestyle influences, and interactions with other hepatic metabolic pathways may modulate GGA homeostasis and cancer susceptibility. Addressing these limitations in future studies will be essential for translating these mechanistic insights into clinically applicable strategies.

In conclusion, the MAOB–GGA–CYP3A4 axis appears to serve as a central regulator of the age-related transition from hepatic homeostasis to hepatocarcinogenesis. MAOB plays a critical role in sustaining endogenous GGA biosynthesis, which contributes to immune-mediated tumor surveillance in the liver. Its age-associated decline constitutes a metabolic vulnerability that coincides with an increased risk of hepatocellular carcinoma. While CYP3A4 may partially compensate for the loss of MAOB activity, this compensation comes with potential drawbacks, including elevated reactive oxygen species generation (Unsal and Belge-Kurutaş, 2017) and reduced efficacy of chemotherapeutic agents due to enhanced drug metabolism (Tian and Hu, 2014; Özkan et al., 2021). Although this review focused on hepatic tumor suppression, recent evidence also suggests broader physiological roles for GGA, particularly in male reproductive health (Tabata et al., 2021; Tabata et al., 2020), highlighting its systemic relevance. As aging populations become increasingly represented among patients with liver cancer, identifying metabolic biomarkers such as GGA may support the development of targeted preventive strategies and inform clinical decision-making in geriatric oncology. A deeper understanding of the temporal dynamics and functional balance within the MAOB–GGA–CYP3A4 axis could thus provide novel insights into age-adapted cancer prevention.
